# Assessing Moral Judgements in Veterinary Students: An Exploratory Mixed-Methods Study from Germany

**DOI:** 10.3390/ani12050586

**Published:** 2022-02-25

**Authors:** Kirsten Persson, Wiebke-Rebekka Gerdts, Sonja Hartnack, Peter Kunzmann

**Affiliations:** 1 Applied Ethics in Veterinary Medicine Group, Institute for Animal Hygiene, Animal Welfare and Farm Animal Behaviour, University of Veterinary Medicine Hannover, Foundation, Bischofsholer Damm 15, Geb.116, 30173 Hannover, Germany; wiebke-rebekka.gerdts@tiho-hannover.de (W.-R.G.); peter.kunzmann@tiho-hannover.de (P.K.); 2 Section of Epidemiology, Vetsuisse Faculty, University of Zurich, Winterthurerstr. 270, 8057 Zurich, Switzerland; sonja.hartnack@access.uzh.ch

**Keywords:** veterinary ethics, ethics education, vignettes, qualitative research, quantitative research

## Abstract

**Simple Summary:**

On the one hand, veterinary ethics is a required part of veterinary education. On the other hand, the success of ethics teaching and the students’ skills concerning judgements in morally demanding situations are hardly evaluated systematically. This article presents an innovative tool to evaluate those skills in veterinary students in a first case of application. One group of students in this case had taken ethics classes, the other had not. The participants were asked to fill in a questionnaire with different scenarios from veterinary practice and answer additional free-text questions. Students who had taken ethics classes did not answer generally different from those students who had not taken ethics classes. However, there were many overall differences between the students’ answers, decisions, attitudes, and explanations. The tool is therefore suggested for further evaluations of ethics teaching and moral judgement skills in veterinary students.

**Abstract:**

Although veterinary ethics is required in veterinary curricula and part of the competencies expected of a trained veterinary surgeon according to the European Association of Establishments for Veterinary Education (EAEVE), knowledge concerning the effects of ethics teaching and tools evaluating moral judgement are scarce. To address this lack of tools with a mixed-methods approach, a questionnaire with three case scenarios presenting typical ethical conflicts of veterinary practice was administered to two groups of veterinary students (one had taken ethics classes, one did not). The questionnaire contained both open-ended and closed questions and was analysed qualitatively and quantitatively. The qualitative part aimed at revealing different argumentation patterns between the two groups, whereas the quantitative part focused on the students’ approval of different roles and attitudes possibly relating to veterinarians. The results showed no major differences between both groups. However, answering patterns suggest a clear diversity among the students in their perception of morally relevant factors and the veterinary profession. Awareness of morally challenging elements of their profession was presented by students of both groups. With this exploratory study, the application of an innovative mixed-methods tool for evaluating the moral judgement of veterinary medical students is demonstrated.

## 1. Introduction

With growing public awareness of the interests and needs of animals, the role of veterinary professionals is increasingly gaining responsibility, albeit rather subtly/implicitly. In an age of companion animals in the literal sense—being (replacements for) family members or other human companions in life [1]—and highly specialised livestock breeds, veterinarians in veterinary practices in Western societies, such as human medical professionals, are equipped with advanced medical facilities and treatment options. Similar to situations in human medicine, veterinarians are therefore regularly faced with ethically challenging contexts [2,3,4,5].

These circumstances should be reflected in veterinary education. Undeniably, professional veterinary ethics is a crucial part of undergraduate study programmes in veterinary medicine, as stated in the EU Directive 2005/36/EC (5.4.1 Study programme for veterinary surgeons, B. Specific subjects, a. Basic sciences: “professional ethics”) and in the “FVE, AWARE & EAEVE Report on European Veterinary Education in Animal Welfare Science, Ethics and Law” (June 2013) [6]. Among the extensive list of Day One Competences of veterinarians is, for example, the ability to “appraise the social context and participate in societal debates about animal welfare and ethics” [7], p. 2 accompanied by the recommendation that “Animal Welfare science, ethics and law should be a core subject, and examinable with the same pass/fail criteria as other core subjects.” [7], p. 3. The implementation of these recommendations has been the subject of recent evaluation on a Europe-wide scale [8], revealing an overall increase in time spent teaching animal (welfare) ethics and the estimation that all Day One Competences are covered or even exceeded by the current curricula. Most evaluations, however, fail to explicitly differentiate between animal welfare science, animal ethics and veterinary ethics. Basically, interdisciplinary animal welfare science comprises natural and social sciences focused on animal farming and husbandry regarding accessing and improving animal welfare [9]. Animal welfare science is not interchangeable with or a synonym of animal ethics for several reasons, not least because the methods and assumptions are mutually criticised [10]. Animal ethics is a domain of philosophical ethics that is neither restricted to certain types of animals nor to certain normative assumptions and, furthermore, not to questions of application in veterinary contexts. Veterinary (medical) ethics, as a discipline comparable to human medical ethics, includes sub-disciplines such as history, philosophy and sociology of veterinary medicine, professional ethics and, in its core, structured reasoning regarding moral norms guiding judgements and decisions in veterinary practice. Although animal ethics and animal welfare science can be valuable assets for a veterinary student or a veterinarian, veterinary ethics in the explained sense present an essential basis for the veterinarian profession and are an indispensable part of any veterinary curriculum [11]. The Learning Objectives, corresponding to the abovementioned Day One Competences of Veterinarians, mirror the broad understanding of veterinary ethics [6], p. 15. In this study, the focus will be primarily on “Personal and Professional Competences/Attributes” (LO 17–23), “Human-Animal Relationships” (LO 25–29) and, to some extent, on “Welfare Legislations, Regulations and Norms” (LO 30–35). In other words, only that part of veterinary ethics that considers the students’ moral attitudes and judgements, including their reasoning and orientations in the process, is studied. At the same time, we acknowledge that teaching veterinary ethics for good reasons includes further dimensions of ethics, too; for example, those covered in the other Learning Objectives.

In contrast to human health professionals’ training, exam regulations for veterinary ethics within veterinary education are by no means standardised, if there are any exams at all. In human health professional training, first attempts to standardise ethics training have been made, but veterinarians have not been included [12]. Thus, whether a newly-approbated veterinarian is well-equipped for making ethically challenging judgements and for decision making has not been the subject of standardised evaluations so far. Furthermore, the tools for testing such skills are not as conveniently available as they are for other theoretical and practical scientific skills in veterinary education [13,14,15,16,17].

While some aspects of ethical skills are potentially disclosed when making decisions and through actions, others might be instilled in a veterinarian’s overarching professional attitude. Veterinarians may have different attitudes in their profession towards their patients and as a result may have different roles in a triangle of tension between the veterinarian, animal owner and the animal [18]. Especially in situations that are experienced as moral conflicts, the veterinarian needs to be aware of the roles he or she can take on and the responsibilities he or she bears, e.g., to work out solutions for these conflicts or to preserve his or her mental health [4,19,20,21]. Veterinarians balance the interests of many actors on an “ethical high wire” [22], p. 15: those of the animals, the owners, society, the environment and also their own interests. Whose interests the veterinarian prioritises is the “fundamental question of veterinary ethics” [23], p. 27. As a consequence of this prioritisation, a veterinarian assumes different roles. Rollin described the “mechanic” and the “paediatrician” as possible roles [23]. Veterinarians can thus take on the role of “service provider” or “salesperson” [24], “animal protector” or “animal advocate” [25,26]. Possibly, veterinary students’ identifications with those roles could be differentiated with orientations such as a patient owner’s autonomy, economic aspects or a patient’s quality of life.

With these general attitudes being coined by professional role models or growing personal work experience, the foundation is potentially already laid during the educational years of a veterinary student. The implementation of ethics courses should present a valuable accompaniment for students during this process.

In 2015, the University of Veterinary Medicine Hannover (TiHo) implemented the first (and until today the only) Chair for Applied Ethics in Veterinary Medicine in Germany [27]. Therefore, the TiHo presents a unique opportunity to evaluate the effect of ethics teaching to veterinary students.

### The Veterinary Ethics Curriculum in Hannover since 2015

The term “applied ethics” is decisive for the teaching and implies “identifying and analysing proposed solutions for ethically relevant cases of conflict and at the same time […] developing and representing one’s own solutions as being more appropriate” [28], p. 6 (own translation)). The interaction between the two principles of bottom-up and top-down, i.e., between concrete individual real-life cases and their individual solutions, alternating with general ethical considerations and standards, is the main guiding principle [27]. This system is called “critical supervision”. Teaching veterinary ethics addresses those situations where students may be confronted with ethical questions. Therefore, ethics is part of the curriculum in at least three of its stages: in the first year, a weekly “Introduction to Professional Ethics” is held, where students are familiarised with the basic content of ethical theories and veterinary professional ethics, in particular also with the “Ethics Code of Veterinarians in Germany” [29] and its recommendations for actions [30]. During an agricultural internship at the university’s Farm for Education and Research, two seminars are held at the beginning and end of the internship focusing on conflicts in industrial livestock farming. In the third year, the series of lectures “Ethics for the Clinic” exemplifies “critical guidance” [27]. Veterinarians from university clinics present examples of conflicting cases and practices and discuss potential solution strategies with the students. Students thus gain insight into real conflicts and can consolidate their points of view and sharpen their judgement. In an accompanying lecture, the cases are reviewed and discussed with the help of ethical tools. There are also cross-year workshops organised, entitled “Practice-Ethics-Practice”. Here, students can discuss with invited veterinarians from the region their individual moral conflicts and experiences in practice and develop generally applicable solutions in accordance with the principle of bottom-up and top-down. Additional seminars and lectures focus on issues such as the killing and slaughter or pain therapy of animals, animals in agriculture, as well as general and specific animal ethics issues and theories, also in the public discourse. In cooperation with the Clinical Skills Lab, nudges are used as thought-provoking short case scenarios to highlight potential sources of moral stress for students. The syllabus in ethics is continuously aligned with the Learning Objectives and Day One Competences of the EAEVE and FVE [6]. Ethics is taught by an interdisciplinary team of veterinarians, agriculturists and humanities researchers with philosophical expertise and philosophers and is headed by a professor of philosophy.

The study presented in this article picks up the above explained lack of tools for evaluation of ethics teaching and suggests a mixture of methods to assess veterinary students’ decision-making and judgement skills in morally demanding situations.

The overarching goal was to present a tool that is applicable on a large scale and appropriate for evaluating the ethics-related day-one competencies of veterinary students. As those competencies cannot be assessed in a multiple-choice exam and as in-depth evaluations such as qualitative interviews are not applicable on a large scale, the aim was to develop an innovative approach to reveal the students’ awareness and their ability to weigh different arguments and perspectives in morally challenging situations. Combining qualitative and quantitative methods with ethical reasoning presents a tripartite approach, each complementing the results of the other two and revealing a multi-perspective access to the research questions.

In the presented study we demonstrate the application of our mixed-methods tool. The small data set generated in this first and exploratory study serves as an example to demonstrate what kind of data can be obtained with the tool and what kind of research questions could be approached. The study’s limited sample size (due to a limited availability of students in the pandemic) does not allow for an in-depth comparative analysis of argumentation patterns regarding essential differences between the two groups of students we built. Therefore, we rather suggest aspects worth analysing and discussing with the help of our tool including potential refinements and additional research questions.

Our prototypical data analysis focusses on two main research questions:

1. Does it make a difference in the students’ moral judgement competencies, argumentation patterns or attitudes whether they had purposefully been exposed to challenges, arguments and judgment criteria in veterinary contexts? If this was the case, differences between those students who attended (multiple) ethics courses and those who did not should be detectable with the empirical approach presented in this article. The selected cohort veterinary students after the four-year scientific-theoretical study component of their education was the first group having the opportunity to attend the above-described revised version of the veterinary ethics curriculum at the TiHo Hannover. Despite the broad spectrum of options, taking ethics classes was facultative for the students, as compulsory electives are additionally offered from several other fields of veterinary medicine. Apart from very few exceptions (e.g., the ethics class accompanying the farm internship), students were, on the one hand, able to avoid having to attend ethics classes in their field of education, or, on the other hand, accumulate lectures, weekend-seminars and in-depth reading courses on a broad range of topics in animal and veterinary ethics.

To address the first research question, in a first step, case scenarios from veterinary practice displaying an ethical conflict and a necessity for a veterinarian to decide were presented to the students.

In order to carve out judgement and decision patterns regarding the first research question, this study systematically asked students to:

- identify stakeholders of different conflicts in veterinary practice;
- show awareness of the complexity of ethical conflicts;
- think about obtaining all necessary information in acute situations; and
- come to a well-founded decision in the given case scenarios.

2. Besides the differences between students who had taken ethics classes and those who had not, the spectrum and type of arguments presented by the students were expected to give insight into the more general (ethical) argumentation patterns of veterinarians. In order to carve out those types of convictions or attitudes, the students’ quantitative answers were attributed to at least one of eight characteristic orientations (O1–O8) that will be further elaborated in the Materials and Methods: Autonomy of the Patient Owner (O1), Financial Problems of the Patient Owner (O2), Quality of Life of the Patient (O3), Own Ethical Attitude (O4), Own Financial Situation (O5), Communication (O6), Animal Welfare Law (O7) and Avoiding a Decision (O8). The aim of the analysis of the Likert scales was two-fold. First, to assess if having taken ethics classes or not could be predicted by the quantitative answers grouped into the eight orientations. The second aim was to assess whether patterns across the eight orientations could be detected, i.e., if students who ranked the owner’s autonomy high also ranked communication high.

That way, the significance of those different factors for the students’ answers was to be analysed quantitatively and discussed in relation to their free-text-answers from the first part of the study. Being an exploratory study, our research aim is, in this step, descriptive and, as such, meant as a basis for further specification, validation and more evaluative research questions. We rather want to focus on the process of how to apply the tool and how to use the data obtained in the process than on the specific results of this first study.

## 2. Materials and Methods

The survey was pilot tested among ca. 15 members of the institute who were not involved in designing or conducting this study but who are teaching ethics classes regularly and partially have a background in veterinary science. Their suggestions regarding wording and authenticity of the presented cases were implemented in the scenarios. The survey was made available online (LimeSurvey GmbH, Hamburg, Germany, www.limesurvey.org, accessed on 19 February 2022) to a 262-student cohort in their fourth year of veterinary education. The call for participation was first sent to the students via email on 14 July 2020. The constraints due to the pandemic made it impossible to gather the students for this purpose, as originally planned, in an exam-like situation and hand out a printed version of the survey. Rather, online participation was facultative, advertised via email. After several email reminders data collection was finished after approx. 16 weeks. The survey was closed on 31 October 2020.

In the survey, three fictional scenarios were presented (see Supplementary Materials File S1 for original version). In all scenarios, a conflict was presented, involving economic aspects, the veterinarian’s understanding of their professional role and ethics, animal welfare/rights aspects and the patient owner’s perspective.

Step 1: Free-text questions

For each scenario, the participants were asked:

1. Which stakeholders are involved in the presented conflict?
2. To what extent is there an ethical conflict?
3. Do you need further information before making a decision in this case?
4. How would you decide if you were the veterinarian?

Step 2: Likert scale questions

After filling in the free-text answers, a list of five to six different statements was presented to the students for each scenario (see Table 1).

On a Likert scale from one (very poor) to nine (very good), they were asked to indicate their agreement with each statement. Each statement was attributed to at least one of eight different orientations (O1–O8) that were presumed to play a role in decision-making for that scenario.

The scenarios:

1. Farm Scenario: “A veterinarian is called to a farm to examine and treat a cow with mastitis. On the farm, she notices that other cows are coughing but the farmer does not want her to examine them. He insists that “it is only the weather” and that the veterinarian should only treat the one cow with mastitis”.

2. Companion Animal Scenario: “A woman and her eight-year-old daughter bring a guinea pig to the veterinarian’s practice. The animal is in urgent need of medical treatment and is suffering from pain. The woman does not want to pay €200 for the required therapy but asks the veterinarian to put the guinea pig down. She wants to buy her daughter a new one for €25. The daughter, however, is devastated”.

3. Jumping Horse Scenario: “A veterinarian is called to a jumping horse who is lame on the left front leg. After a thorough examination, he orders treatment and rest. The owner explains that the horse is scheduled to compete in two days’ time and asks the veterinarian to suppress the lameness by administering an analgesic. When the veterinarian points out that competition stress under these conditions could lead to permanent tendon damage, the owner replies that he has taken out life insurance for the horse”.

The orientations are:

- O1: Autonomy of the Patient Owner (PO): Taking into account and, in case of doubt, prioritising the autonomy of the PO over other aspects.
- O2: Financial Problems of the PO: taking into account economic constraints of the PO and considering them as substantial for decision-making.
- O3: Quality of Life (QOL) of the Patient: Considering the patient’s quality of life and putting it above other factors.
- O4: Own Ethical Attitude: Consistently representing one’s own position and not moving away from it when begged by patient owners.
- O5: Own Financial Situation: Accepting own economic losses if a situation calls for it, especially if the owner can/does not want to pay.
- O6: Communication: Striving for communicative solutions or emphasising the necessity of a communication process.
- O7: (Animal Welfare) Law: Invoking the legal framework/referring to animal welfare law as a basis or reason for a decision.
- O8: Avoiding a Decision: Postponing or avoiding making a decision.

The orientations are meant as suggestions rather than a complete, closed list of influential factors. They were discussed and specified in the above-described pilot phase of the study. Some reflect the well-known field of tension of the veterinary profession (e.g., financial constraints), while others resemble Beauchamp and Childress‘ guiding principles in medical ethics (autonomy, patient’s welfare) that often have to be weighed against each other during decision-making processes in human medicine [31]. During the analysis, the list of orientations was open to additions and modifications on the basis of the students’ free-text answers in Step 1.

Veterinary and human medical ethics, despite sharing various common challenges and arguments, vary in a number of crucial aspects [31], such as the complex triangular relationship between the veterinarian, the client/patient owner and the patient itself [26], financial constraints or the general accessibility of animal preferences and welfare. Acknowledging the attempts to establish principles in veterinary ethics [32,33,34], the presented study starts with the assumption of several, normatively not (yet) unified approaches and personal orientations in veterinary ethics.

Furthermore, the offered orientations are not meant to serve as a normative ranking from desirable to less-desirable attitudes, although veterinary ethicists would most likely agree that not all eight orientations are preferable results of their teaching efforts.

Finally, the students were asked to indicate which ethics classes (from the above presented curriculum) they had attended during their education.

In order to describe the data statistically, the mean and standard deviation of the Likert scale responses are presented separately for students with and without ethics classes.

Answers were exported into Microsoft^®^ Excel (Version 2012). Two groups of students were identified: those who had taken at least one of the ethics classes from the list (“with ethics”, WE) and those who had not (“no ethics”, NE). For each question, the answers for the two groups were imported in MAXQDA (2020, Analytics Pro Semester, Software for qualitative data analysis, VERBI Software. Consult. Sozialforschung GmbH, Berlin, Germany), separately.

**Figure 1:**
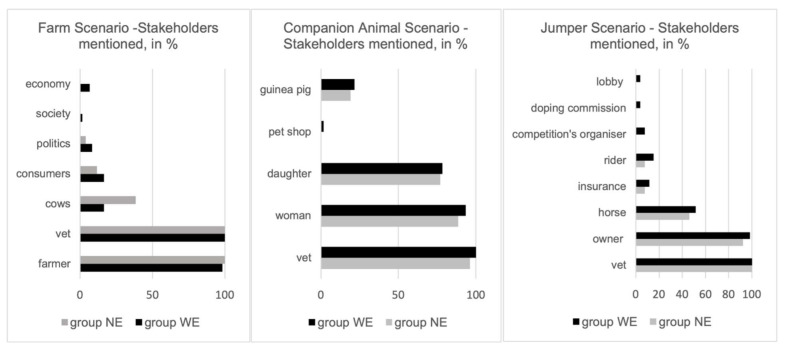
Stakeholders mentioned in the three scenarios, both groups. Answers in %.

**Figure 2:**
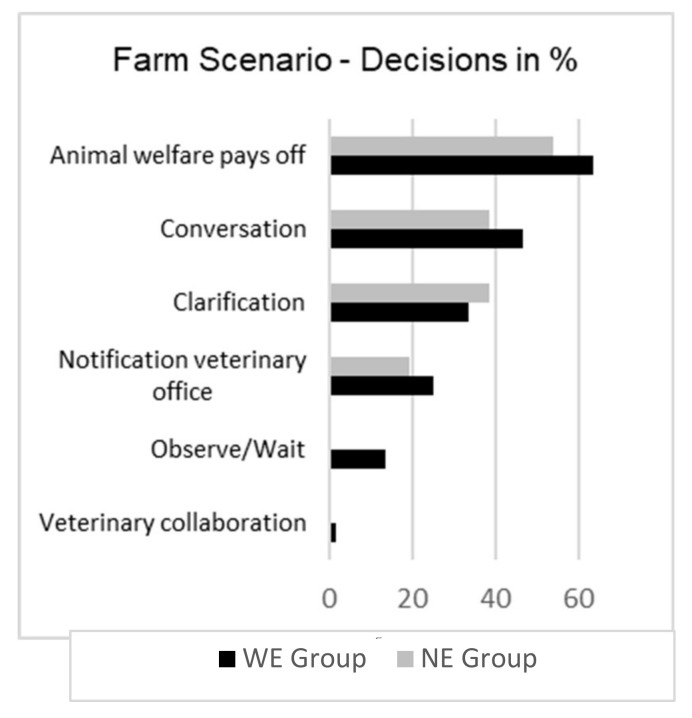

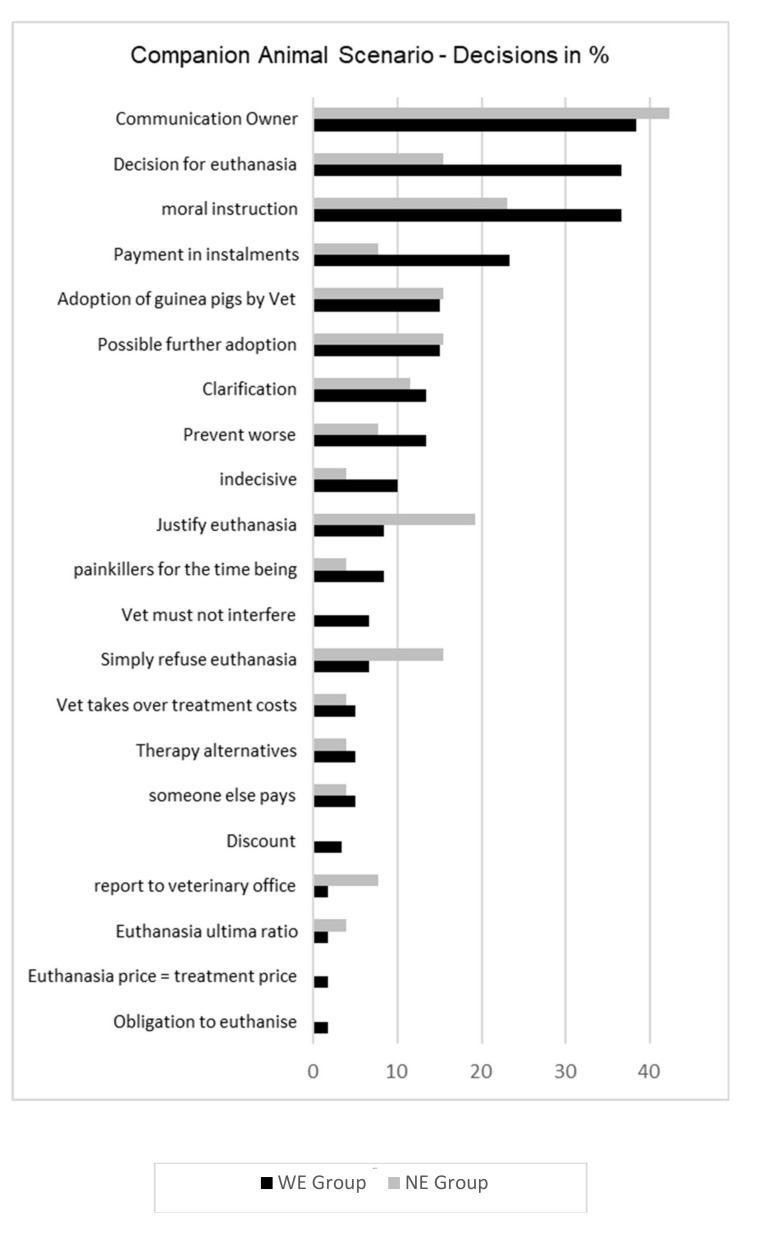

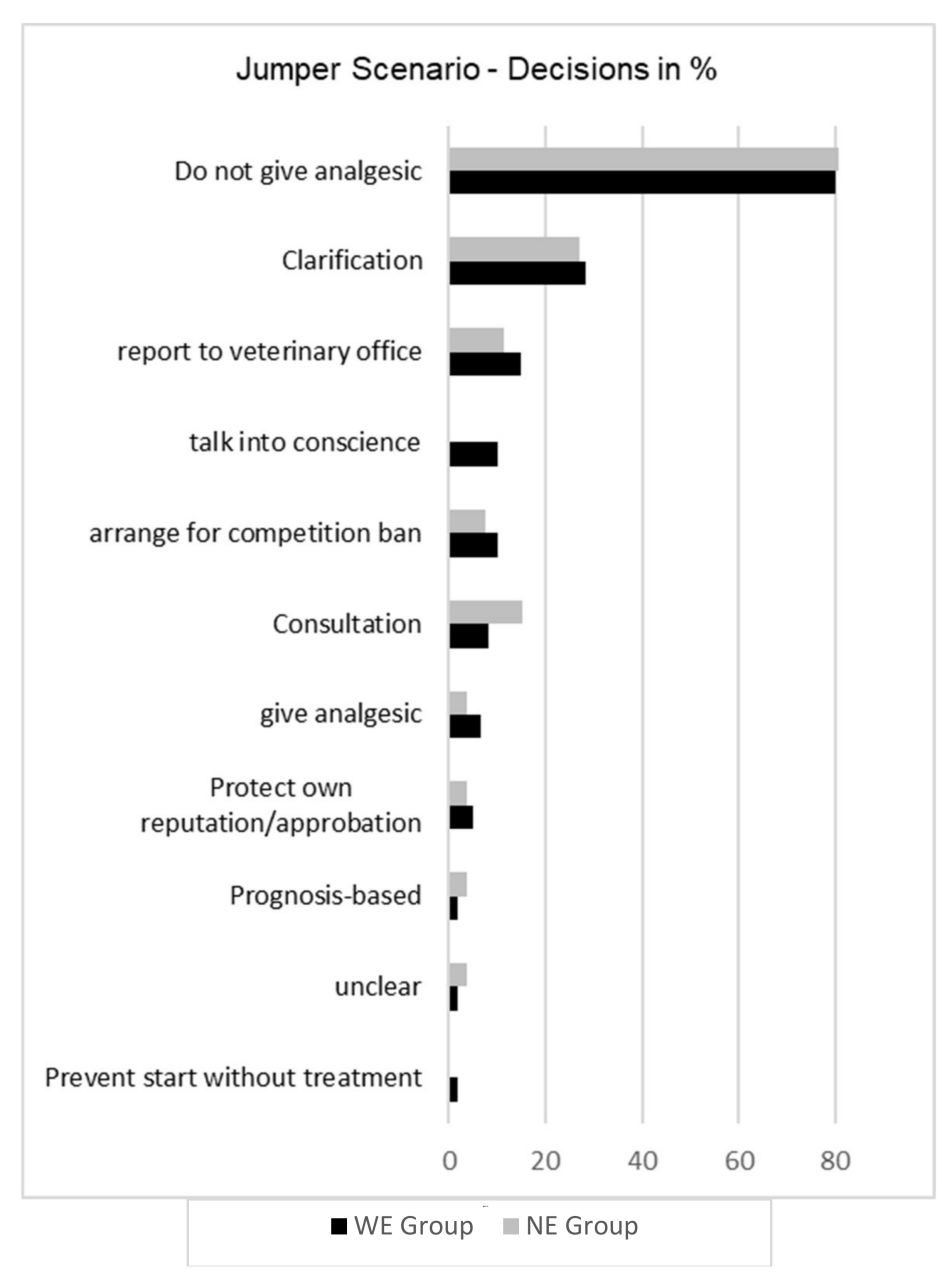
Decisions for all three scenarios, both groups. Answers in %.

The free text answers were analysed with MAXQDA by one member of the team (KP). First, data were separated in two sub-sets for the groups WE and NE. Afterwards, thematic coding was done comparatively: themes and arguments were collected for each free-text question of each scenario, going through the students’ short answers repeatedly. Then, themes and subthemes were refined and sorted so that recurring connections between aspects, similar wordings and justification patterns were identified for the texts of both groups. Figure 1 and Figure 2 were produced in Microsoft^®^ Excel (version 2102). Core quotes, i.e., statements that either represented an opinion that was frequently mentioned or an exceptional, remarkable point of view, were collected and are presented in the analysis.

For the Likert scales, we constructed a Learning Vector Quantisation (LVQ) model in R version 4.0.4 [35] with the packages caret [36] and e1071 [37], with a ten-fold cross-validation. The aim of the LVQ, a supervised classification algorithm, was to estimate separately for each of the eight orientations which statement from a subset of statements might be relevant to a particular orientation and be used to classify students with and without ethics education. The importance of each variable was estimated with the varImp() command by an ROC curve analysis. The results are presented in the form of a ranking of the Likert scales in predicting having taken ethics classes or not.

For the second aim, we applied a clustering by k-means designed for dependent data with the kml package [38]. We chose the number of parturitions (from two to six) based on the Calinski and Harabasz criteria from Genolini [39] and from Kryszczuk [40] and ran the k-means 50 times with different starting conditions.

Since we might have created an artificial clustering by including the same Likert scale for several orientations, in a subsequent analysis, we reduced the number of responses included so that every Likert scale was just included once for the k-means analysis (Supplementary Materials Figure S2).

## 3. Results

### 3.1. General Free Text Results

The return-rate was ca. 33% (*n* = 86). Out of the 86 participating students, 61 (70.9%, 95%CI (60.8;79.7)) indicated that they had taken ethics classes. The three scenarios generated divergent results regarding the spectrum of mentioned aspects, the heterogeneity of decisions and the congruency with expected answers:

Although no major differences could be detected between the groups’ WE and NE perspectives and judgements in the free-text answers, a pattern throughout all three scenarios emerged concerning the WE Group, which presented a slightly broader range of aspects (stakeholders, information needed, decisions) in their responses. For an overview of answering patterns, and, particularly, a comparison between the two groups WE and NE, see Figure 1 and Figure 2.

Several recurring thoughts and arguments could be found in all three scenarios. Especially the ambivalent relationship to economic constraints regarding therapeutic decisions was frequently raised, revealing a broad spectrum of attitudes, for example:

- A conception of veterinarians as service providers:

“*On the other hand, the farmer authorises the veterinarian with a certain examination and treatment by presenting a patient; the veterinarian cannot treat without having been authorised to do so*.” (WE Group, Farm Scenario, question “to what extent is there an ethical conflict?”).

- A conception of veterinarians as business owners and, therefore, being focussed on securing their income:

“* […] economic interest,* i.e., *possibly losing the customer if one contradicts him*.” (NE Group, Farm Scenario, “to what extent is there an ethical conflict?”),

“*If no one can or wants to pay for the treatment, you can’t permanently be charitable yourself*” (NE Group, Companion Animal Scenario, “How would you decide?”).

- A conception of veterinarians as “the animals’ advocates” and, thus, putting life and welfare of the patient above the client’s or the veterinarian’s economic preferences:

“*I see this as a general duty of a veterinarian to protect the animals, even on a farm you depend on economically.*” (WE Group, Farm Animal Scenario, “How would you decide?”),

“*I would operate on the guinea pig myself as far as my finances allow and then look for a new home within the practice/among other patient owners.*” (NE Group, Companion Animal Scenario, “How would you decide?”).

- The widespread strategy to convince the client that the medically indicated decision is also the economically most advantageous one:

“*Try to convince the farmer that healthy animals can also perform better.*” (NE Group, Farm Animal Scenario, “How would you decide?”),

“* […] in cows, lung diseases and reduced well-being affect milk yield, etc., and one should aim for a healthy herd in the long term. Thus, veterinary costs should be seen more as investments than as costs.*” (WE Group, Farm Animal Scenario, “How would you decide?”),

“*Reference to profitability. To miss a tournament, now, but to be able to win many more later will certainly generate more money*” (NE Group, Jumping Horse Scenario, “How would you decide?).

In some statements, the argument is explicitly used in a strategic way, as it is perceived most convincingly: “*In doing so, she should make clear to the farmer that sick animals produce less output and thus mean a loss of profits. In most cases, this financial loss calculation is more likely to reach the ears of livestock farmers*.” (WE Group, Farm Animal Scenario, “How would you decide?”).

### 3.2. Scenario-Specific Free-Text Results

Specific findings for each scenario are presented with exemplary quotations from the free-text answers.

#### 3.2.1. Farm Scenario

The Farm Scenario displayed a broad range of perspectives and judgements. On the one hand, the participants explained their sympathy for the farmer’s situation, especially for his economic and practical constraints. They referred to both his professional responsibility and his expertise regarding his herd. Regarding the veterinarian, participants described her inner conflict between professional duty/ethos and the role as a service provider for her client. Different strategies to compromise between the farmer’s and the cows’ requirements and needs were raised, both perspectives being weighed differently.

Participants indicated as stakeholders—besides the obvious, farmer and veterinarian—the cows, consumers and, rarely (<10%), also politics, society and economy (see Figure 1). When describing the ethical conflict, more than half of the WE Group mentioned the farmer’s economic challenges on the one hand and animal welfare on the other hand.

“*On the one hand, the veterinarian cannot leave the other cows untreated from an ethical point of view, as these animals also have a right to treatment. On the other hand, it cannot be expected that the farmer runs into financial difficulties because he cannot afford the treatment and possibly faces such financial problems that his existence is endangered in the process.*” (WE Group).

However, more than 40% of both groups also described a conflict between the general task or duty of a veterinarian and the possible choices for the veterinarian in this case, especially regarding customer loyalty. This aspect was more frequently mentioned by the NE Group.

“ […] *The veterinarian is obliged to alleviate or remedy pain, suffering and damage if possible. Everyone who keeps an animal is obliged to care for it according to its needs and to prevent pain, suffering and damage. Purely economic reasons cannot be considered a reasonable cause under the Animal Welfare Act. Veterinary Professional Code, Animal Welfare Act <-> economic interest. On the other hand, the farmer commissions the veterinarian to perform a certain examination and treatment by presenting a patient the veterinarian cannot treat without having been authorised to do so.*” (WE Group).

One participant in the WE Group even suggested that:

“*If no compliance can be achieved at all, the veterinarian should discontinue his veterinary care on this farm and, depending on the severity of the problem, the local veterinary office must be informed.*”

On the one hand, the WE Group more frequently ascribed economic problems to the farmer, overall, and several participants offered a sympathetic perspective:

“*The farmer is also important, and you can’t ask him to ruin himself financially. However, animal welfare is also just as important*” (WE Group).

On the other hand, the NE Group was inclined to call the farmer’s situation a refusal to pay:

“*The veterinarian has the problem that the farmer does not want to have the sick animals treated by her for financial reasons, although it would be appropriate.*” (NE Group).

Regarding the question how they would decide in that situation (see Figure 2), the answering patterns in both groups differed mainly in one way: only members of the WE Group suggested waiting or observing as their (preliminary) decision.

“*For the moment, let the matter settle. If necessary, inform the farmer of possible causes and explain why an early examination to detect and treat any disease that may be present and may even be spreading in the herd would be economically better than waiting until it is unavoidable. If the farmer cannot be convinced to have the animals examined immediately, another look should be taken at the follow-up examination of the cow that was actually treated. You can also ask the farmer to get back to you should the cough not subside in a few days, the symptoms get worse or more animals are affected.*” (WE Group).

As mentioned above, most decision-related answers (>50%) in both groups, however, suggested a communication strategy based on the argument summed up as “animal welfare pays off”.

#### 3.2.2. Companion Animal Scenario

The entire spectrum of attitudes and solutions was presented in the Companion Animal Scenario: different main conflicts were identified, either emphasising the economic aspect, the disagreement between mother and daughter or more general challenges concerning euthanising a per se treatable animal. Participants asked for further information about the specific disease, age and general health condition of the guinea pig, procedures and side effects of the therapy and alternative therapy options. Consequently, decisions for or against euthanasia were explained and justified in several ways by the participants, including, for example, communicational aspects, calling the veterinary office, adopting the animal or euthanising it as requested. Most participants (>90%) agreed that the main stakeholders in this conflict were the veterinarian and the mother. However, the daughter was also mentioned by more than 70% in both groups. Approximately 20% considered the guinea pig a stakeholder.

The conflict was described as a trade-off (intrinsic value of the guinea pig vs. monetary value, expressed by what the owner is [not] willing to pay) or as a more general question in how far an animal with a treatable disease should or may be euthanised.

“*The guinea pig’s illness would be treatable but costs the pet owner too much money. This presents the conflict between animal life and costs.*” (WE Group).

“*There is the possibility of treatment. Therefore, euthanasia is not necessary and is carried out in this case for financial reasons.*” (WE Group).

More abstract concepts such as responsibility or the right to live were more likely to be raised by the WE Group, whereas justifications for a euthanasia were discussed to a larger extent by the NE Group.

Hence, thoughts regarding a justification of euthanasia were also more often raised as reasons for their decisions by the NE Group. In this group, a higher percentage of participants would choose to simply refuse euthanasia or inform the veterinary office:

“*I would definitely not euthanise the animal that is treatable.*” (NE Group).

In the WE Group, a much larger part of the participants decided to euthanise the animal (37% compared to 15% in the NE group) and justified their decision regularly with preventing even greater harm:

“*or the animal would die miserably at home, even if I give an analgesic temporarily, that would not be a nice solution*” (WE Group).

Approximately 30% made their decision dependent on further information regarding the age and health of the guinea pig. Additionally, the WE Group was more concerned about the decision of a second veterinarian in case they sent the patients away:

“*The client will probably find another colleague who will euthanise the animal as she wants. I don’t think it’s right to euthanise the animal only because someone else will do it. You should set an example and stand by your convictions. If everyone did that, such owners would have fewer chances.*” (WE Group).

Several participants reflected on weighing up the (presumed) quality of life against the (presumed) benefit of a prolonged life or the loss through death for the guinea pig, or, in fact, animals in general:

“*If the opinion does not change and no further rehoming is possible, euthanise to avoid further suffering. An animal will probably suffer more from chronic pain than from the dying process.*” (NE Group, “how would you decide?”).

As one of the reasons to treat but not to euthanise, the Animal Welfare Law was referred to by participants of both groups. The German Animal Welfare Act prohibits killing an animal without reasonable cause (§1). Some students judged euthanasia of a treatable animal as not in line with that law:

“*Law: You may not euthanise an animal without reasonable cause.*” (NE Group, “to what extent is there an ethical conflict?”).

“*I would point out to the mother that she is responsible for the health of the animal in accordance with the Animal Welfare Act and that you can’t just put an animal down like that.*” (NE Group, “how would you decide?”).

Suggesting possible solutions, many students offered financial compromises and other measures “*such as instalment payments to alleviate some of the financial burden.*” (WE Group).

Four students even considered fully covering the costs, “*but only if the mother handed over the animal*”. (WE Group, “how would you decide?).

#### 3.2.3. Jumping Horse Scenario

For the Jumping Horse scenario, the open-ended answers were rather homogeneous, as the overall judgement regarding the situation seemed clear: the client’s demand and his attitude towards the animal were considered out of question. The veterinarian should not give an analgesic to render the animal ready for the competition.

Nearly all participants agreed that the veterinarian and the horse’s owner were the main stakeholders in the conflict. Half of the participants also considered the horse a stakeholder. Additionally, the rider, the horse’s insurance, organisers of the competition, the doping commission or the jumping horse lobby were mentioned on several occasions.

The conflict was identified between animal welfare and the patient owner’s sporting and financial ambitions. The WE Group also frequently (>70%) mentioned insurance fraud as part of the ethical conflict compared to 15% in the NE Group.

As their decision regarding further proceedings was dependent on the medical consequences for the animal, participants claimed to require more information regarding the details of the injury and the prognosis under different circumstances.

“*What exactly is the lameness? The extent/degree of lameness is decisive for the type and success of therapy*” (WE Group, “Further Information?”).

Approximately 80% of the participants explicitly expressed their reluctance to inject the analgesic, mainly because of the danger of long-term harm to the jumping horse. Furthermore, they referred to doping being illegal and in violation of animal welfare, which is why some were also worried about the veterinarian’s approbation. Similar to the other two scenarios, communicational aspects were emphasised as an important part of the veterinarian’s job:

“*In this case, I would not perform any treatment at the request of the owner, which could lead to long-term harm to the animal. With the expression “the horse has a good life insurance” he shows that he does not care about the welfare of the animal and profit is important to him. I would not support this in any case. In addition, I could imagine that with such a “treatment error”, my license to practise medicine would be at risk. Finally, I would try to explain this to the owner again and bring him to his senses. I see the attitude of the owner as problematic: He will probably just call the next veterinarian and ask him to inject the analgesic…*” (WE Group, “How would you decide?”).

### 3.3. Results Likert Scales

The responses in the form of agreeing with the statements, on a 9-point Likert scale with increasing agreement, are presented in Table 2, separately for students with and without ethics classes. Highest in agreement ranked the statements S17, S10, S11, S5 and S1. Lowest agreements were expressed for the statements S16, S13, S4 and S12. Differences of at least 0.5 between students with and without ethics were present for the statements S9, S17, S10, S8 and S2.

Table 2: For each of the 17 statements, the mean and standard deviations are presented for students both with and without ethics classes. In subsequent analyses, the statements were grouped together in eight different orientations, with some of the statements contributing to more than one orientation.

The LVQ model assessed the importance of each statement relevant for a specific orientation. The statements S8, S9, S15, S16 and S17 were consistently ranked high, with an importance of at least 70% (see also Supplementary Figure S1).

**Figure 3:**
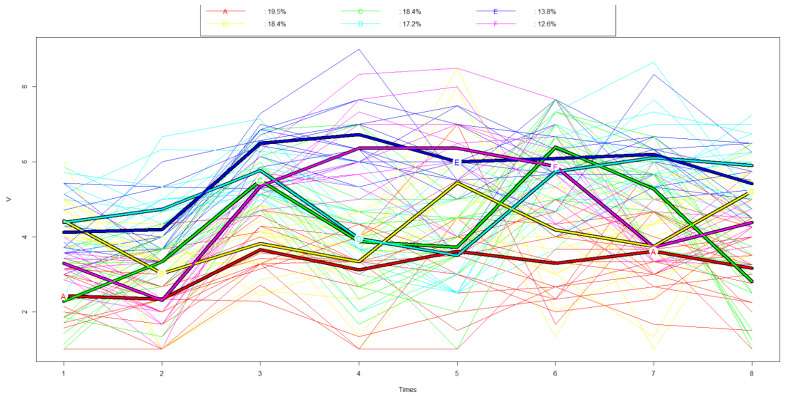
Based on the k-means clustering, six distinct clusters (A to F) were detected displaying different trajectories across the eight orientations.

With the aim of assessing whether a pattern exists in the agreement with the eight orientations, a k-mean clustering was performed and resulted in the best parturition of six patterns (A to F, Figure 3, Table 3). Apparently, students who scored high in QOL (O3), Attitudes (O4), Veterinarian Finances (O5), Communication (O6) and Law (O7) were grouped together in Cluster E, whereas those which scored low, were assigned to be in cluster A. Students in Cluster B were characterised by a high score for Autonomy (O1), Finances Vet (O5) and Avoidance (O8), while all other orientations scored below the mean. Students in Cluster C scored particularly high in QOL (O3), Communication (O6) and Law (O7) and had the lowest score in Avoidance (O8). Students in Cluster D shared the high scoring in QOL (O3), Communication (O6) and Law (O7) with Cluster C, but scored additionally high in Autonomy (O1), Finances PO (O2) and even had the highest score in Avoidance (O8). Students belonging to cluster F scored particularly high in Attitudes (O4), Vet Finances (O5), with most of the other orientations being close to the overall mean or even below.

## 4. Discussion

The discussion is to be understood as an example of how data obtained with the developed mixed-methods tool can be discussed against the FVE&EAEVE learning objectives, legal framework, findings from the literature and general ethical considerations. In the limitations section, we acknowledge that our sample size was insufficient to find significant or, qualitatively speaking, meaningful results. The tendencies and slight differences and the setup of the tool give reason, though, to expect potential between-group differences for larger samples.

Case vignettes have been used for teaching and evaluation purposes elsewhere [14,41]. The innovative aspect of our mixed-methods tool is the combination of free-text questions giving the students the opportunity to develop their own judgements and arguments and, in a second step, a list of alternatives that represent different general attitudes of veterinarians (orientations). Beyond data generation this method has a potential for inducing reflection. Letting the students contrast their own solution with the given statements might already give them feedback regarding their position on the broad spectrum of orientations. Additionally, comparing the free-text answers to the list gives us the opportunity to extend and revise the tool and refine the spectrum of orientations. The data set presented in this article, for example, suggests the addition of the “pragmatic orientation”: convincing the client of a treatment option by arguing that they would also economically benefit from it.

The data discussion is also meant to show the applicability of our tool independently of the specific curriculum at the TiHo as we follow the learning objectives valid for all veterinary training institutes in the EU.

The first focus of our study lay on differences in answering patterns between those students who attended ethics courses and those who did not. With the three different scenarios, we tried to cover a broad range of typical challenges and conflicts in veterinary practice situations (see for example Batchelor 2012), accompanied by the equally broad spectrum of decisions or solutions regarding those [2]. The approach relates to the equally broad spectrum of Day One Competences and corresponding Learning Objectives [LO] stated by the FVE & EAEVE ([6], p. 15). Rather than the students’ knowledge on analytical frameworks like the Five Freedoms (LO 1–5, p. 15) or the students’ ability to access the animal’s biological functioning and welfare (LO 6–16), this study examines their skills in the fields of “Personal and Professional Competences/Attributes” (LO 17–23), “Human-Animal Relationships” (LO 25–29) and, to some extent, of “Welfare Legislations, Regulations and Norms” (LO 30–35); in brief, the competences relevant for veterinary medical ethics. Some of the LO will be referred to in more detail in the following discussion.

Based on our analysis, for all scenarios and questions, no major differences could be found between the two groups in a quantitative analysis. However, in the qualitative analysis, there are some subtle but interesting findings:

The WE Group came up with more stakeholders, factors contributing to the conflict, necessary additional information they required, and also a wider spectrum of choices/decisions. This pattern emerged in all three scenarios (see Figure 1 and Figure 2), giving reason to assume that this effect is not solely due to the difference in group size (WE: *n* = 61, NE: *n* = 26). Rather, it suggests advances regarding different LO. A student that “Recognise the diversity of functions and uses of animals within society” (LO 25) and is able to “describe and debate the different ethical views on animals” (LO 26) will present a differentiated answer regarding, for example, the mother’s and the daughter’s perspectives on their companion animal but also regarding the farmer’s ambivalent attitude towards his animals being coined by both the economic value and the individual bond. Our results are also in line with the prudent suggestion, only made within the WE Group, to wait until the following visit and observe the case rather than acting immediately. Furthermore, the fact that students in WE Group were less inclined to simply refuse euthanasia and would rather decide on having the guinea pig put down (see Figure 2) suggest a capacity captured in LO 28: “Examine the underlying values that justify the rules and norms regarding animal welfare and protection”. Beyond defending the unquestioned protection of animal life, they weigh up the animal’s suffering and prospective quality of life, including the circumstances and limitations of its care in the family, against the benefit of prolonged life and come to differentiated, well-explained conclusions. Admittedly, there is no clear demarcation line, just a difference in frequency: both types of attitudes can be found in both groups.

Despite all efforts to determine two distinct groups regarding their ethics education, the separation is to some extent arbitrary: the difference between a student in the WE Group and one in the NE Group might be one class of, for example, farm animal ethics. We were not able, nor was it our intention, to quantify the degree of being taught in ethics within the WE Group. One reason for the hardly pronounced differences between the results for both groups in both the qualitative and the quantitative part of the study can therefore be attributed to the heterogeneity within and the blurry demarcation between both groups regarding the factor “having taken ethics classes”.

Even if the distinction between the two groups had been more pronounced, other factors outside the veterinary education related to previous training, social and cultural norms and past events might also have shaped the moral judgement and behaviour. Among those influencing factors, a much-discussed candidate is the theory of the “hidden curriculum” [17]. The idea that, in addition to regular ethics classes, veterinary medical ethics is taught implicitly during courses in other disciplines and especially when learning from experienced practitioners who embodied a certain moral attitude, is one of the reasons that veterinary medical ethics—as a separate discipline with academic infrastructure—had difficulties being established [42]. The idea that messages or habits that are not part of the formal curriculum could be transported via the hidden curriculum [43] might be mirrored in some of the students’ unexpected answering patterns in our study: the idea of convincing clients of the necessity of medically indicated treatments by emphasising solely the economic advantages (and not stating any responsibility) is clearly not a strategy systematically taught within the formal curriculum. Thus, neither was this type of answer expected by the study designers, who are all involved in teaching veterinary ethics, in the free-text answers nor was a statement offered that covered this attitude in the Likert scales. Nevertheless, the frequency of raising this aspect (more than 50%, see Figure 2) suggests that students are somehow exposed to this argumentation pattern during their veterinary education. Briefly and exemplarily, this example serves to point out the challenges created by the intertwining of formal and hidden curriculum: while in the given example of coughing cows on a farm, where it seems appropriate to state that the medically indicated and economically wise solutions converge, there are cases in which compromised animal welfare does not correlate with a decrease in economic output. Especially with formalised veterinary medical ethics being a rather newly established part of the curriculum, its teaching goals could be undermined by influential experienced veterinarians who did not attend veterinary ethics courses during their education.

After all, even a more robust sample size might have suggested the conclusion that there is no meaningful difference in moral judgement and argumentation patterns as tested in this study between students who had taken ethics classes and those who had not. The question to which extent ethics courses in general or particular classes influence the students’ moral reasoning and decision making is, among other things, due to the influential factors outside classrooms, as elaborated above, which are challenging to investigate. Tools like the here-presented approach can only provide answers within the scope of their limited research questions. In case of this study, it is plausible to assume that a regular decision making and justification training with similar scenarios during ethics classes will, at least, lead to rather consistent orientation patterns for each student and to well-founded argumentations. Considering a specific example, however, gives reason for doubts: students at the TiHo are repeatedly trained in weighing the animal patient’s, the patient owner’s and the veterinarian’s interests in decision-making processes in veterinary practice. However, our results suggest that most students did not consider the animal patients as stakeholders in any scenario. Indeed, discussing case studies with ethically challenging scenarios is part of many lectures and seminar at the TiHo, but there is no systematic investigation regarding the extent of the students’ habituation to this type of task. Instead of concluding that ethics courses are not influential it could be claimed that their standing in the curriculum and their frequency are too low, and the students do not take them seriously enough, all of which could be modified by re-structuring the curriculum.

One of the core issues when talking about ethically difficult and morally stressful situations in veterinary practice is euthanasia. While the presented Companion Animal Scenario also displayed a financial conflict, many students picked up fundamental issues regarding end-of-life decision in veterinary practice. Some simply referred to the legal framework, as killing animals without reasonable cause is prohibited in Germany. Others, though, discussed more fundamental moral questions, namely, if a veterinarian should euthanise an animal if it was not medically indicated, or, if ending an animal’s life painlessly is presumably more in the animal’s interest than keeping it alive under unclear conditions regarding its care and quality of life. It has been suggested elsewhere [44] that a more profound education regarding the philosophical basis of euthanasia could be essential for veterinary professionals to individually find a coherent ethical framework of reasoning and justifications and, thereby, to prevent moral stress. The participants in our study clearly indicate an awareness for the ethical conflicts beyond what is legally regulated. Still being students, they already confirm a demand for that part of their education in veterinary ethics.

Conflicts concerning financial aspects are well known to veterinary students and broadly identified in all three scenarios. In human medicine, medical costs may be covered by insurance. In veterinary medicine, the client’s willingness to pay is a decisive factor when it comes to more expensive treatment options in veterinary practice. Some students apparently differentiate between the obligation to pay in the case of a companion animal owner and the economic constraints of a livestock owner. On the one hand, the majority of them suggest convincing the woman with the guinea pig to pay as there is no “reasonable cause”, as required by German animal welfare law, for euthanasia. After all, there are treatment options and she, as an owner of a companion animal, bears the responsibility for the animal’s life and well-being. On the other hand, the farmer is supposed to be convinced with the main argument that animal welfare pays off; i.e., it would be worth investing some of his limited resources to be financially better off in the end–with the “by-product” of improved animal welfare. This argumentation pattern obscures whether the students’ basic attitude is motivated more by pragmatic or ethical reasoning.

This leads to the second research question of our study, regarding different types of attitudes and convictions between and within our groups of participants. The heterogeneity of the answers in the free-text questions already suggests a diversity of attitudes of the study participants regarding their role as veterinarians. The results of the Likert scales display this finding even more pointedly (see Table 1 and Table 2). To some statements, especially in the Companion Animal Scenario, the WE and NE groups did not agree to the same extent. However, larger differences could be detected between individual students, also within the same group. The answering patterns, therefore, did not predict if a student had taken ethics classes.

Since due to the special circumstances in distance-learning, the sample size was limited, the initial plan to rely on regression models for assessing the differences between students with and without ethics classes was abandoned. As with the rule of thumb that ten “cases” and ten “non-cases” are needed to assess each risk factor (which would be our statements), with 26 students without ethics classes (the cases), we would only be allowed to analyse two or three statements. Therefore, we decided to use an exploratory, rather hypothetical generating approach with the lvq models and the kml clustering, and not a confirmatory analysis. Similar to the qualitative analysis, in the lvq models, no clear difference between the groups could be found, but the level of agreement with some statements pertaining in particular to the economy, welfare and communication could potentially be used to predict the attendance of ethics classes.

The kml clustering aimed to detect specific patterns in agreeing with the statements. The veterinarian who identifies with a role as a service provider would emphasise their clients’ autonomy, including their willingness to pay. Those students who were inclined to score high on the orientation “Autonomy” (see Table 2) therefore rather agreed to respect the PO’s wishes in the Jumping Horse Scenario, to try and solve the conflict via communication in all three scenarios but to give in if the PO insists on their point of view. Showing an understanding for economic constraints, they also had the tendency to want to treat the guinea pig for free.

Those students who rather sided with the animal patient and scored high on the orientation “Quality of Life” agreed to a large extent to refer to the Animal Welfare Act, to refuse treatment if there was a risk of harming the animal and to adopt the animal in the Companion Animal Scenario. Again, the pragmatic attitude to use successful arguments for a certain intention is revealed in this pattern. Referring to the authority of law rather than stressing their personal or professional judgement might seem more convincing from the students’ perspective. Actual direct references to the Animal Welfare Act in veterinary practice present an interesting subject for further empirical research to contrast these expectations.

The orientation “Attitude” suggested that a veterinarian explains his/her own and very clear attitude to the client and therefore refuses to treat the patients if their requirements conflict with the veterinarian’s basic moral principles or attitude. The foundations for such a mindset could either be laid by a thorough theoretical education or be acquired by experience. Again, further research might shed light on the underlying principles of veterinarians’ decisions and judgements.

Another facet of the students’ general attitudes pointed towards their own economic interests as veterinarians. Having their own business, they need to consider their financial losses if they either lose clients or give discounts/treat patients for free.

In particular, the economic perspective and the veterinarian being paid by the patient owner and not the animal patient, is at the core of typical veterinary ethical dilemmas. This precludes, for example, the complete adaptation of Beauchamp and Childress principles—here, typically, the autonomy of the animals is ill-defined. Furthermore, in this case, well-established tools to evaluate moral reasoning based on Kohlberg’s approach and further developed by Verrinder et al. (DIT) are not (yet) able to provide guidance [14,15,16]. We decided to include a qualitative part that allowed us to detect unexpected findings. That would not have been possible with a German version of a solely quantitative questionnaire based on Verrinder’s DIT test, building on Kohlberg’s normative approach of moral development. Acknowledging the diversity and dynamics of influences on moral judgements, we suggest a non-hierarchical set of orientations rather than a ladder-shaped framework promoting the ultimate goal of moral development. The expansion of the set of orientations is considered an ongoing process throughout the application of our mixed-methods tool.

Further research questions that might be addressed with our tool at different institutions are, for example: Is it a certain class that makes a difference in the students’ attitudes? Is it important to take classes throughout the study programme to be reminded of ethical questions? Are students with a certain normative framework (orientation) more likely to profit from ethics classes? Do the orientations change with more experience/years of study?

The presented analysis is based on a comparatively low number of samples and the qualitative and quantitative analyses were each conducted by only one member of the team. Yet, we consider both the sample size and the presented analysis sufficient to demonstrate the application of our mixed-methods approach regarding the evaluation of moral judgement in veterinary students. In a more comprising follow-up study focused on the results rather than on the method, a larger sample would be needed to, firstly, be able to report more about the whole student cohort of interest; secondly, be able to compare two groups regarding a larger number of variables; and, thirdly, compare the findings to comparable research results from the literature. Nevertheless, valuable insight was gained regarding both a new method and the evaluation of the students’ day-one skills in veterinary ethics.

## 5. Conclusions

Presenting an exploratory study, this article is, beyond the obtained data, meant to offer an example of how to evaluate the ethics-related day-one skills of veterinary students. With the exploratory approach of combining free-text questions, to reveal the students’ awareness of complexity, stakeholders, the main issues and potential solutions of ethical conflicts, with a number of scaled potential responses, to analyse the students’ judgements regarding a given choice of influential factors, a comprehensive large-scale tool is suggested here that needs further refinement in the future. Data analysis already provided some suggestions for revision and expansion of the tool.

## Figures and Tables

**Table 1:** The statements presented to the students.

Scenario	Statement
1: Farm Scenario	S1. The motto is wait and see. Dr. Huf-Schmidt only treats one cow and takes a look at the other animals the day after next during the treatment check.
	S2. Animal welfare should come first. Dr. Huf-Schmidt explains that the other cows also need treatment and that she will only treat the one cow if she is allowed to treat the others.
	S3. According to the [German] Animal Protection Act, it is forbidden to cause pain, suffering or harm to an animal without reasonable cause. Dr. Huf-Schmidt should explain this and threaten to file charges if the farmer continues to refuse treatment.
	S4. Informed decisions of patient owners should not be questioned. Dr. Huf-Schmidt therefore only treats the one cow.
	S5. The economic constraints must be taken into account. Dr. Huf-Schmidt therefore offers the farmer a “bulk discount” when treating the other cows.
	S6. The right communication is crucial. Dr. Huf-Schmidt explains in detail the arguments in favor of treating all animals, but gives in if the farmer does not agree after all, so as not to lose him as a customer.
2. Companion Animal Scenario	S7. The informed decision of the patient owner (PB) takes precedence. The veterinarian should comply with her wishes and euthanize the animal.
	S8. The Animal Protection Act states that an animal owner must pay for the care of the animal. The veterinarian should inform the PB about her duties towards the animal and euthanize the animal only in case of subsequently ordered animal restraint.
	S9. The euthanasia demanded by the PO is not ethically justifiable-curative treatment without payment would not be economically justifiable. The veterinarian should refuse euthanasia and send the family away.
	S10. The welfare of the guinea pig comes first. The veterinarian should take the animal into care and treat it without charging the costs to the PO. Subsequently, it should be passed on via an animal welfare organization.
	S11. It should be possible to convince the PO with arguments. The vet should try with a conversation and only give in if she cannot be convinced.
	S12. All needs must be considered. The veterinarian should therefore explain to the daughter that he will exceptionally treat the guinea pig free of charge if the mother does not want to pay.
3. Jumper Scenario	S13. The informed decision lies with the PO. Dr. Knecht-Weber therefore complies with the request of the PO and administers the analgesic.
	S14. The demand of the PO is ethically not justifiable. Dr. Knecht-Weber therefore refuses the requested analgesic without further discussion.
	S15. This can be clarified in a conversation with well-founded arguments. So Dr. Weber-Knecht tries to convince the owner of the treatment and the protection of the horse and gives the analgesic only if the PO really does not give in.
	S16. The PO’s (economic and prestige) interest in participating in the competition is understandable. Dr. Weber-Knecht agrees with the owner that this is a one-time exception for the important competition and administers the analgesic.
	S17. The measure demanded by the PO violates the animal welfare law. Dr. Knecht-Weber informs the owner about his duties towards the animal and threatens to report him to the responsible veterinary office if he allows the horse to start at the competition.

**Table 2:** Descriptive statistics of the 17 statements.

Statement		Without Ethics	With Ethics	Orientations
	Mean (SD)	Mean (SD)	Mean (SD)	
S1 WaitAndSeeFarm	5.78 (1.9)	5.84 (1.9)	5.75 (1.9)	O8
S2 TreatAllFarm	4.12 (2.3)	3.72 (2.03)	4.28 (2.4)	O3, O4,
S3 AnimalWelfareActFarm	4.29 (2.4)	4.52 (2.3)	4.20 (2.4)	O7
S4 ClientAutonomyFarm	2.37 (1.9)	2.48 (1.9)	2.33 (1.9)	O1
S5 DiscountFarm	5.79 (2.4)	5.72 (2.4)	5.82 (2.4)	O2, O3, O5
S6 CommunicationFarm	5.45 (2.2)	5.2 (2.2)	5.56 (2.3)	O1, O6
S7 ClientAutonomyCompanion	3.28 (2.4)	3.52 (2.6)	3.18 (2.4)	O1
S8 AnimalWelfareActCompanion	4.27 (2.3)	3.76 (2.2)	4.48 (2.3)	O3, O7
S9 SendClientAway	3.42 (2.6)	2.60 (2.1)	3.75 (2.7)	O4, O8
S10 TakeOverCompanion	6.00 (2.3)	5.56 (2.5)	6.18 (2.2)	O3, O5
S11 CommunicationCompanion	5.92 (2.5)	5.56 (2.8)	6.06 (2.4)	O1, O6
S12 FreeTreatmentCompanion	2.49 (1.8)	2.52 (1.6)	2.48 (1.9)	O2, O3, O5, O6
S13 ClientAutonomyJumper	1.86 (1.5)	1.68 (1.1)	1.93 (1.7)	O1
S14 RefusalJumper	5.42 (2.8)	5.16 (3.0)	5.52 (2.7)	O3, O4,
S15 CommunicationJumper	3.81 (2.3)	3.48 (2.2)	3.95 (2.4)	O1, O6
S16 EconomyJumper	1.70 (1.2)	1.88 (1.2)	1.62 (1.2)	O1, O2
S17 AnimalWelfareActJumper	6.89 (2.3)	6.32 (2.3)	7.11 (2.2)	O3, O7

**Table 3:** The six clusters were obtained by k-means clustering using the kml package.

	Total	Cluster A	Cluster B	Cluster C	Cluster D	Cluster E	Cluster F
	Mean (sd)	Mean (sd)	Mean (sd)	Mean (sd)	Mean (sd)	Mean (sd)	Mean (sd)
O1	3.44 (1.22)	2.42 (0.75)	4.43 (0.68)	2.28 (0.91)	4.38 (1.03)	4.12 (0.87)	3.30 (0.45)
O2	3.31 (1.23)	2.33 (0.90)	3.02 (0.95)	3.33 (0.75)	4.73 (0.91)	4.19 (0.87)	2.3 (0.78)
O3	5.00 (1.22)	3.65 (0.69)	3.82 (0.77)	5.51 (0.62)	5.77 (0.69)	6.50 (0.54)	5.35 (0.41)
O4	4.35 (1.76)	3.12 (1.06)	3.33 (1.05)	3.87 (1.58)	3.95 (0.94)	6.72 (1.00)	6.36 (1.06)
O5	4.62 (1.71)	3.62 (1.45)	5.44 (1.49)	3.72 (1.06)	3.50 (1.10)	6.00 (1.15)	6.36 (1.43)
O6	5.16 (1.62)	3.29 (1.07)	4.19 (1.50)	6.39 (0.95)	5.73 (1.09)	6.08 (1.10)	5.88 (1.02)
O7	4.74 (1.51)	3.61 (1.11)	3.75 (1.30)	5.27 (0.99)	6.11 (1.07)	6.19 (0.87)	3.73 (0.77)
O8	4.41 (1.51)	3.16 (1.07)	5.22 (0.93)	2.81 (1.18)	5.90 (0.88)	5.42 (0.76)	4.39 (0.74)

## Data Availability

The data presented in this study are available on request from the corresponding author. The data are not publicly available in order to keep the anonymity of our (small number of) participants.

## Supplementary Materials

The following supporting information can be downloaded at https://www.mdpi.com/article/10.3390/ani12050586/s1, File S1: Questionnaire administered via Lime Survey, Figure S1: Importance plots resulting from the LVQ (Learning Vector Quantisation) models, Figure S2: Each Likert scale was included only once in the k-means analysis.
