# Supplementary material for: Assessing Moral Judgements in Veterinary Students: An Exploratory Mixed-Methods Study from Germany
Source: Animals (Basel). 2022 Feb 25;12(5):586. doi: 10.3390/ani12050586 (PMC8909237; doi:10.3390/ani12050586)
Supplement: Supplementary file 1 [file animals-12-00586-s001.zip › animals-1574368 Figures S1-S2.pdf]

O1 Autonomy PO

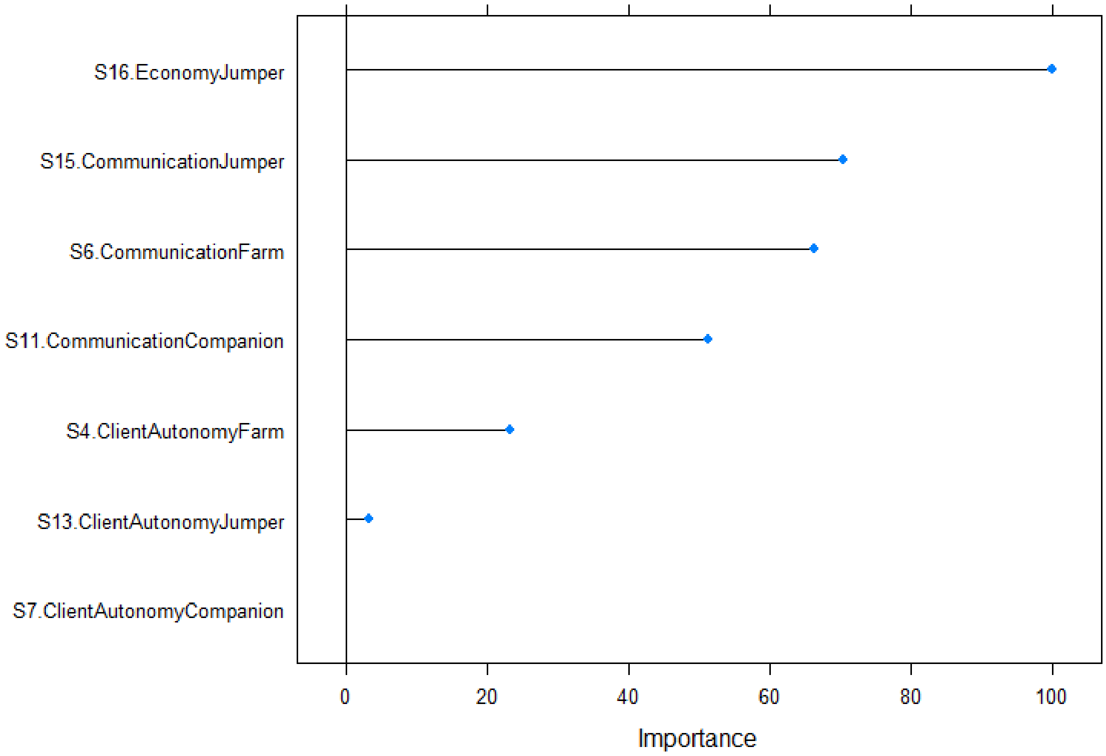

Finances PO O2

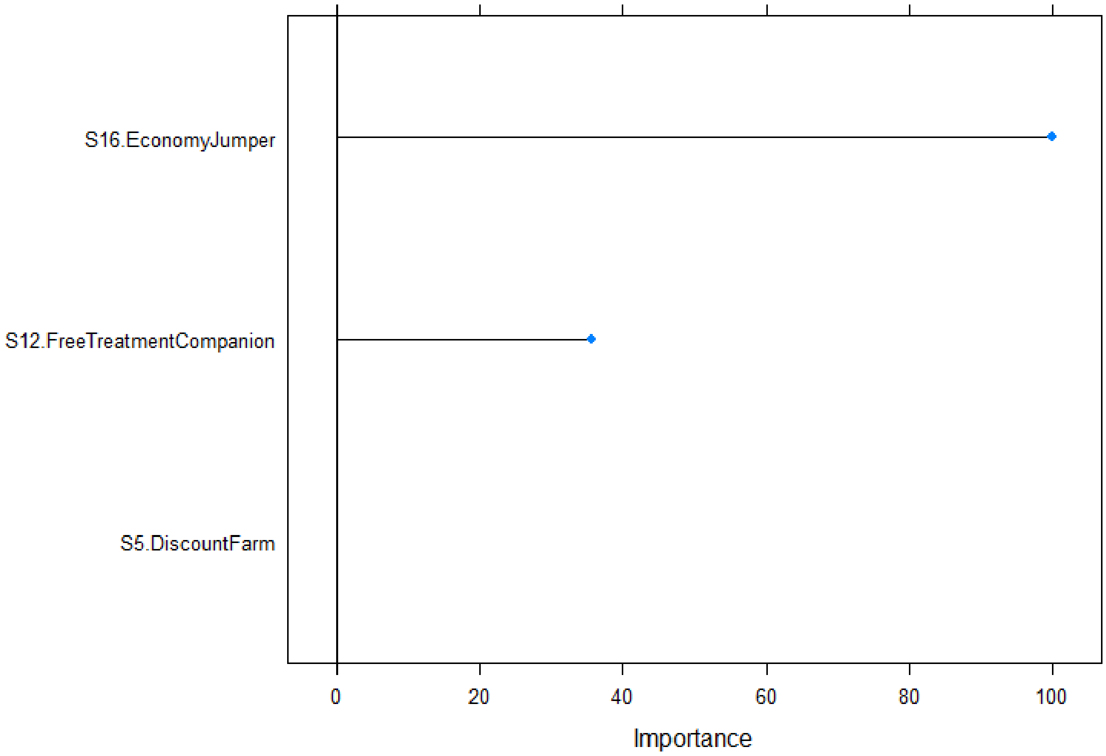

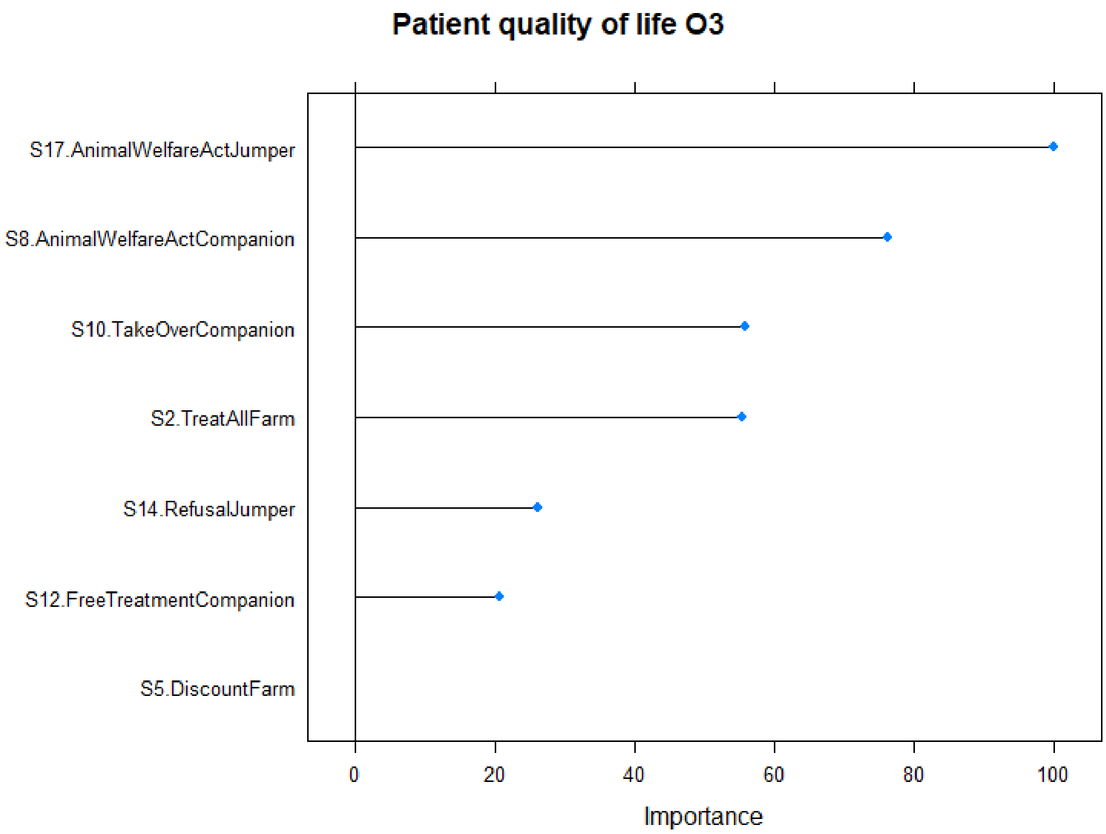
Patient quality of life O3
S17.AnimalWelfareActJumper
S8.AnimalWelfareActCompanion
S10.TakeOverCompanion
S2.TreatAllFarm
S14.RefusalJumper
S12.FreeTreatmentCompanion
S5.DiscountFarm
0
20
40
60
80
100
Importance

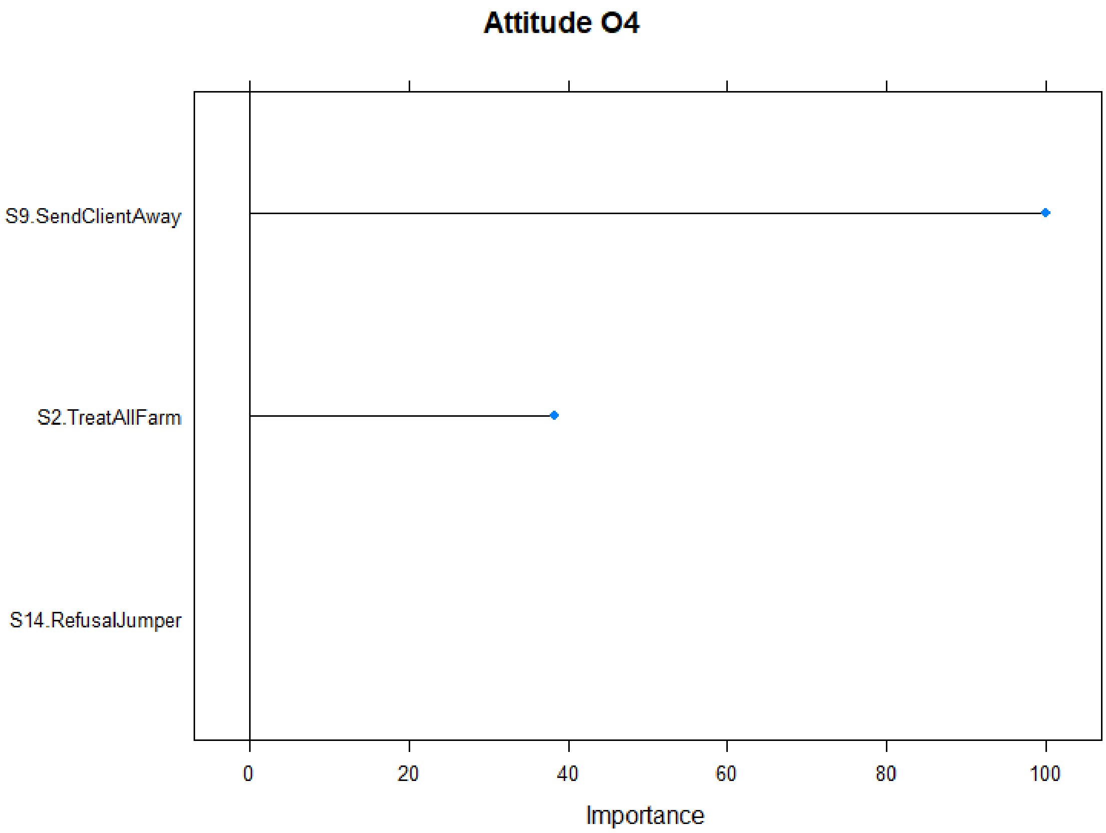
Attitude O4
S9.SendClientAway
S2.TreatAllFarm
S14.RefusalJumper
0
20
40
60
80
100
Importance

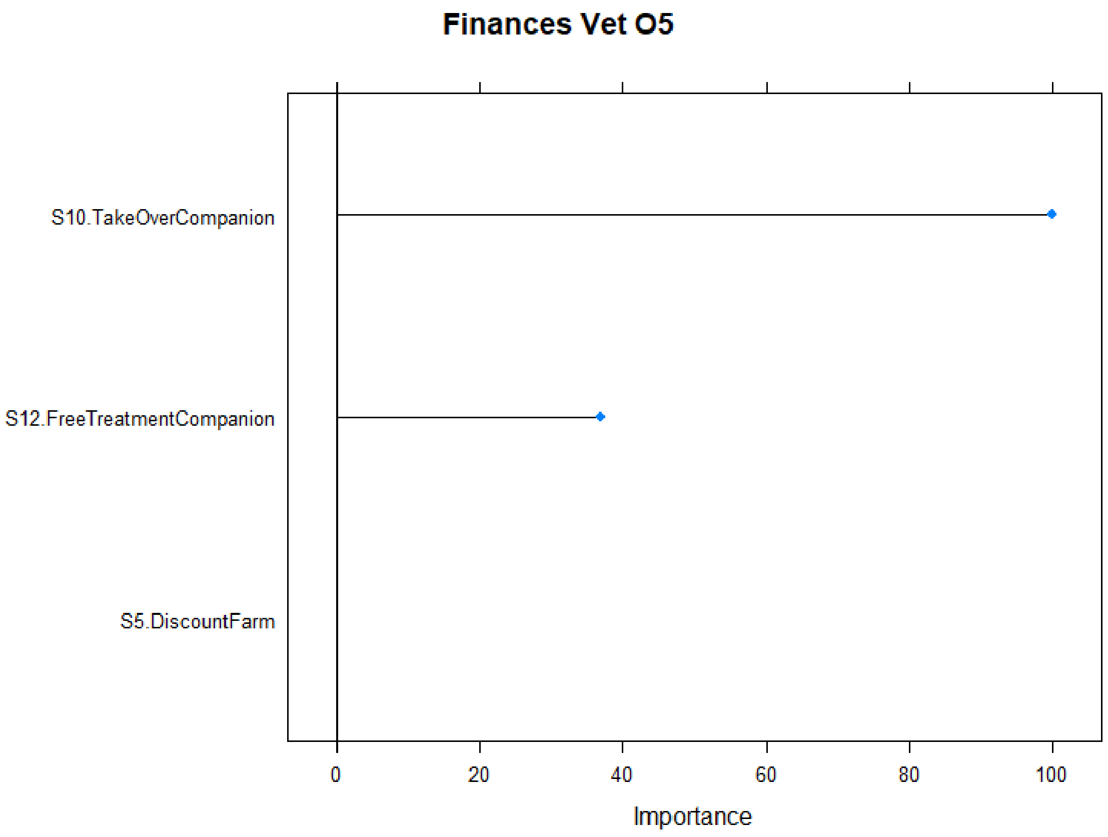
Finances Vet O5
S10.TakeOverCompanion
S12.FreeTreatmentCompanion
S5.DiscountFarm
0
20
40
60
80
100
Importance

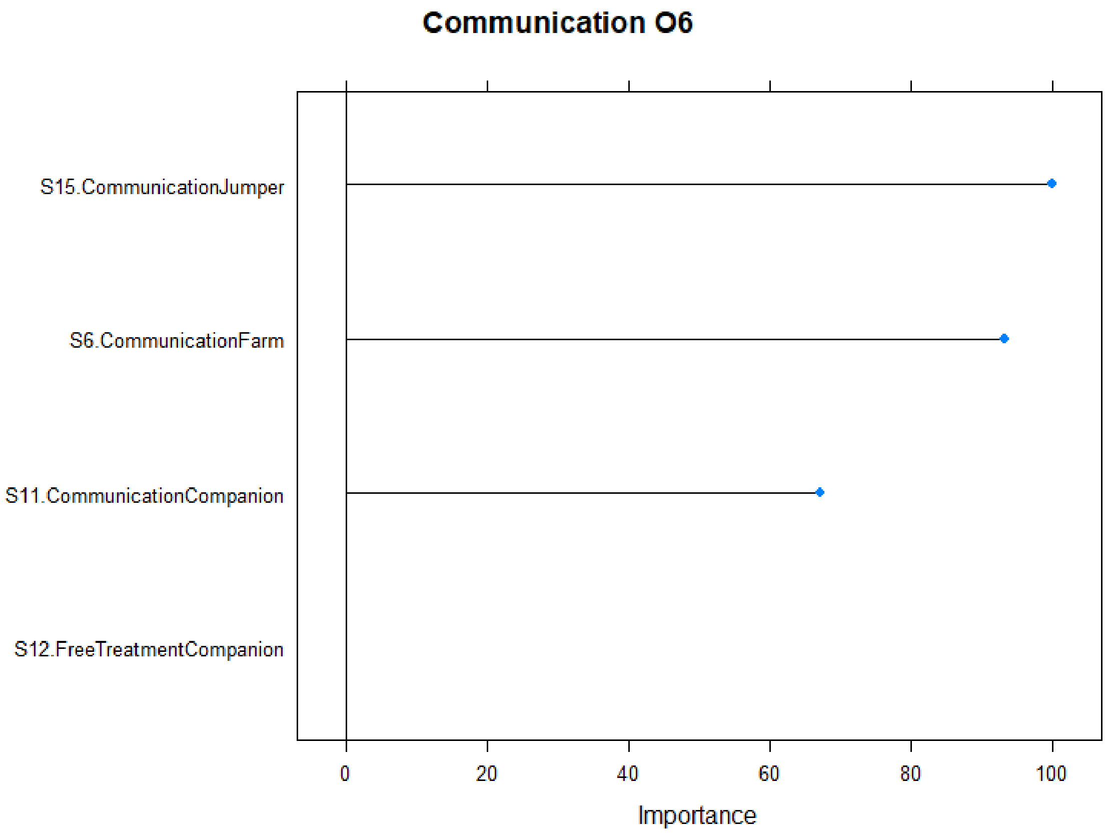
Communication O6
S15.CommunicationJumper
S6.CommunicationFarm
S11.CommunicationCompanion
S12.FreeTreatmentCompanion
0
20
40
60
80
100
Importance

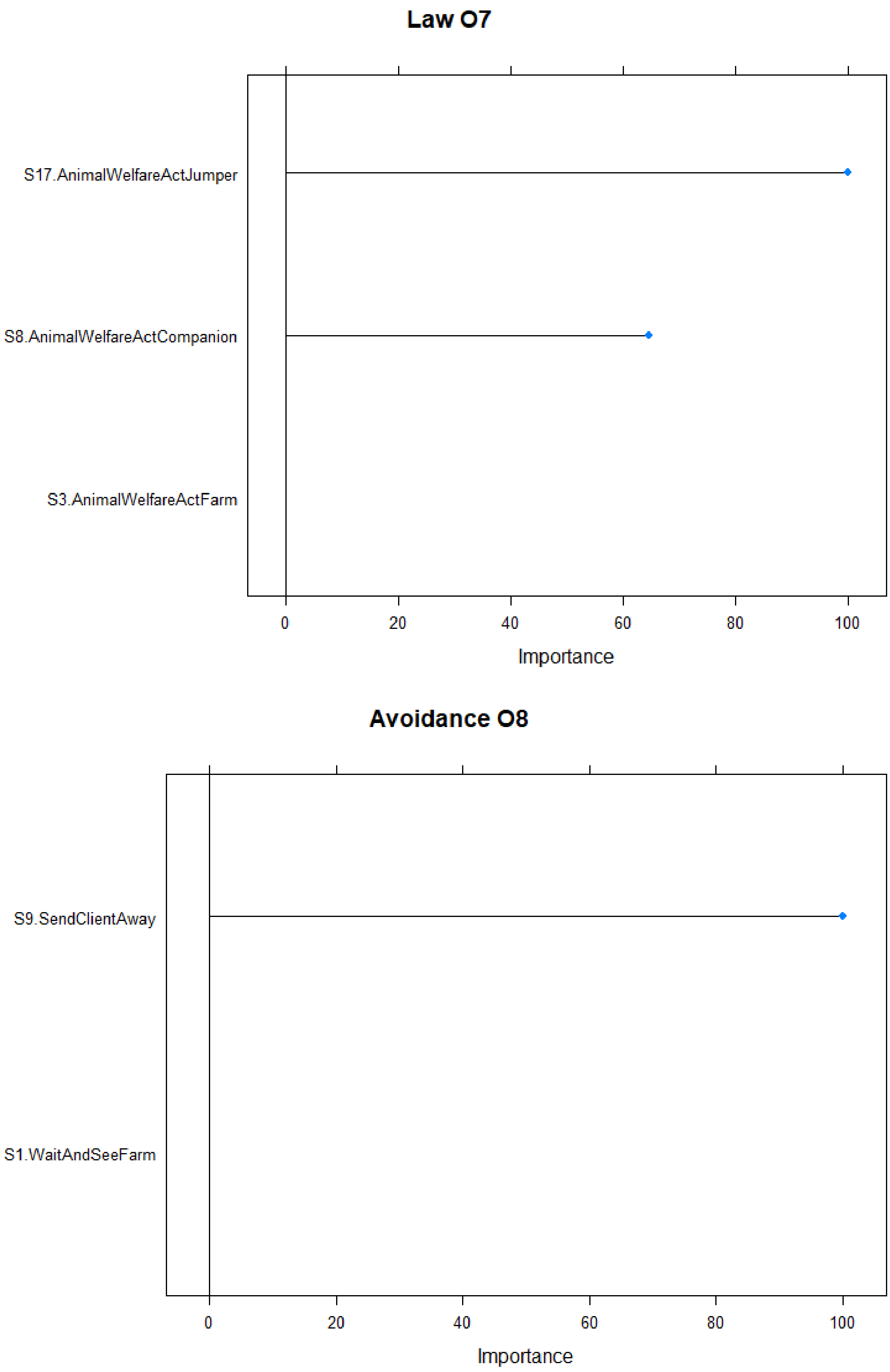

Figure S1. portance plots resulting from the LVQ (Learning Vector Quantisation) models.

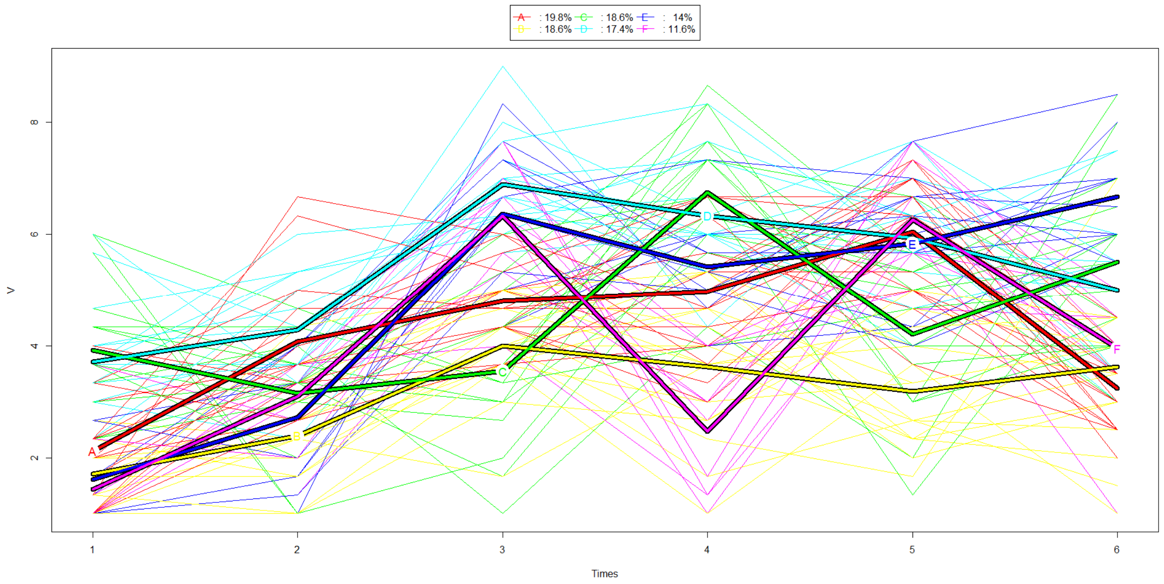

Figure S2. Each Likert scale was included only once in the k-means analysis.
