# Supplementary material for: Assessing Moral Judgements in Veterinary Students: An Exploratory Mixed-Methods Study from Germany
Source: Animals (Basel). 2022 Feb 25;12(5):586. doi: 10.3390/ani12050586 (PMC8909237; doi:10.3390/ani12050586)
Supplement: Supplementary file 1 [file animals-12-00586-s001.zip › animals-1574368-supplementary File S1.pdf]

„Was denken Sie?“ – Ethische Urteile in der Tiermedizin

Liebe Studierende,

im Rahmen des vom Bundesministerium für Bildung und Forschung finanzierten Projektes FERTHIK II führt die AG Angewandte Ethik in der Tiermedizin eine Studie zu ethischen Urteilen bei Studierenden der Tiermedizin durch.

Ihr Jahrgang wurde für diese Studie ausgewählt und wir sind darauf angewiesen, dass Sie sich möglichst vollzählig beteiligen, um aussagekräftige Ergebnisse zu erzielen.

Im Folgenden werden Sie gebeten, sich vier fiktive Fälle aus dem tierärztlichen Praxisalltag durchzulesen und dazu Stellung zu nehmen. Bitte lesen Sie die Texte sorgfältig durch, damit Ihnen keine wichtigen Informationen entgehen.

Bitte beantworten Sie die Fragen in der vorgegebenen Reihenfolge - ein Zurückwechseln zu bearbeiteten Aufgaben ist nicht möglich. Bitte antworten Sie in freien Textfeldern in Stichpunkten! Die Bearbeitung der Umfrage wird ca. 30 Minuten in Anspruch nehmen. Nun geht es los...

In dieser Umfrage sind 23 Fragen enthalten.

Fall 1

Tierärztin Frau Dr. Huf-Schmidt wird zu einem kleineren Milchviehbetrieb gerufen, wo sie eine Kuh mit schwerer Mastitis behandeln soll. Das Tier hat Fieber und die Milchbildung ist stark zurückgegangen. Frau Huf-Schmidt sieht auf dem Hof weitere Kühe, die stark husten. Der Landwirt sagt, das sei halt das Wetter. Sie solle lediglich der einen Kuh wieder zur üblichen Milchleistung verhelfen. Unter den aktuellen ökonomischen Zwängen könne er nicht für jedes Wehwehchen eine tierärztliche Behandlung veranlassen.

1. Wer ist an dem Konflikt beteiligt (Stakeholders)?
2. Inwiefern liegen in diesem Fall ethische Konflikte vor?
3. Welche Informationen sind wichtig für eine Entscheidung? Benötigen Sie weitere Informationen?
4. Wie sollte Frau Dr. Huf-Schmidt weiter vorgehen? Begründen Sie!

Fall 2

Eine Frau kommt mit ihrer 8-jährigen Tochter und deren Meerschweinchen in die Tierklinik. Das Tier leidet an einer behandelbaren Krankheit, aber die zeitaufwändige

Therapie würde etwa 200€ kosten. Die Frau sagt, sie möchte die Kosten nicht übernehmen, und bittet darum, das Tier zu euthanasieren. Sie will ihrer Tochter für 25€ ein neues Meerschweinchen kaufen. Die Tochter ist sichtlich aufgelöst. Das Meerschweinchen leidet an starken Schmerzen und es besteht akuter Handlungsbedarf.

1. Wer ist an dem Konflikt beteiligt (Stakeholders)?
2. Inwiefern liegen in diesem Fall ethische Konflikte vor?
3. Welche Informationen sind wichtig für eine Entscheidung? Benötigen Sie weitere Informationen?
4. Wie würden Sie als Tierärztin/Tierarzt entscheiden? Begründen Sie!

Fall 3

Tierarzt Dr. Knecht-Weber wird zu einem Springpferd gerufen, das am linken Vorderbein lahmt. Nach eingehender Untersuchung ordnet er eine Behandlung und Schonung an. Der Besitzer erklärt, dass das Pferd in zwei Tagen bei einem Wettkampf antreten soll, und fordert ihn auf, die Lahmheit mit der Gabe eines Analgetikums zu unterdrücken. Auf den Hinweis, dass eine Wettkampfbelastung unter diesen Bedingungen zu einem bleibenden Sehnenschaden führen könnte, entgegnet er, das Pferd habe eine gute Lebensversicherung.

1. Wer ist an dem Konflikt beteiligt (Stakeholders)?
2. Inwiefern liegen in diesem Fall ethische Konflikte vor?
3. Welche Informationen sind wichtig für eine Entscheidung? Benötigen Sie weitere Informationen?
4. Wie sollte Herr Dr. Knecht-Weber entscheiden? Begründen Sie!

Skalen Fall 1

Denken Sie an den ersten Fall zurück:

Tierärztin Frau Dr. Huf-Schmidt wird zu einem kleineren Milchviehbetrieb gerufen, wo sie eine Kuh mit schwerer Mastitis behandeln soll. Das Tier hat Fieber und die Milchbildung ist stark zurückgegangen. Frau Huf-Schmidt sieht auf dem Hof weitere Kühe, die stark husten. Der Landwirt sagt, das sei halt das Wetter. Sie solle lediglich der einen Kuh wieder zur üblichen Milchleistung verhelfen. Unter den aktuellen ökonomischen Zwängen könne er nicht für jedes Wehwehchen eine tierärztliche Behandlung veranlassen.

Bitte wählen Sie die zutreffende Antwort für jeden Punkt aus:

Statement	1 (sehr schlecht	2	3	4	5	6	7	8	9 (sehr gut)
Erst einmal abwarten, ist die Devise. Frau Dr. Huf-Schmidt behandelt nur die eine Kuh und wirft bei der Behandlungskontrolle am übernächsten Tag einen Blick auf die anderen Kühe.	O	O	O	O	O	O	O	O	O
Das Tierwohl sollte an erster Stelle stehen. Frau Dr. Huf- Schmidt erklärt, dass die anderen Kühe auch behandlungsbedürftig seien und sie die eine Kuh nur behandeln werde, wenn sie auch die übrigen behandeln dürfe.	O	O	O	O	O	O	O	O	O
Laut Tierschutzgesetz ist es verboten, einem Tier ohne vernünftigen Grund Schmerzen, Leid oder Schäden zuzufügen. Frau Dr. Huf- Schmidt sollte dies erläutern und mit einer Anzeige drohen, falls der Landwirt die Behandlung weiter verweigern sollte.	O	O	O	O	O	O	O	O	O
Aufgeklärte Entscheidungen von	O	O	O	O	O	O	O	O	O

Patientinnenbesitzer*innen sollten nicht infrage gestellt werden. Frau Dr. Huf-Schmidt behandelt also nur die eine Kuh.									
Die ökonomischen Zwänge müssen berücksichtigt werden. Frau Dr. Huf-Schmidt bietet daher dem Landwirt einen „Mengenrabatt“ bei der Behandlung der anderen Kühe an.	O	O	O	O	O	O	O	O	O
Die richtige Kommunikation ist entscheidend. Frau Dr. Huf- Schmidt legt ausführlich die Argumente dar, die für die Behandlung aller Tiere sprechen, gibt aber nach, falls der Landwirt doch nicht einwilligt, um ihn als Kunden nicht zu verlieren.	O	O	O	O	O	O	O	O	O

Skalen Fall 2

Bitte denken Sie zurück an den zweiten Fall:

Eine Frau kommt mit ihrer 8-jährigen Tochter und deren Meerschweinchen in die Tierklinik. Das Tier leidet an einer behandelbaren Krankheit, aber die zeitaufwändige Therapie würde etwa 200€ kosten. Die Frau sagt, sie möchte die Kosten nicht übernehmen, und bittet darum, das Tier zu euthanasieren. Sie will ihrer Tochter für 25€ ein neues Meerschweinchen kaufen. Die Tochter ist sichtlich aufgelöst. Das Meerschweinchen leidet an starken Schmerzen und es besteht akuter Handlungsbedarf.

Bitte wählen Sie die zutreffende Antwort für jeden Punkt aus:

Statement	1 (sehr schlecht	2	3	4	5	6	7	8	9 (sehr gut)
Die aufgeklärte Entscheidung der Patientenbesitzerin (PB) geht vor. Der Tierarzt sollte ihrem Wunsch nachkommen und das Tier euthanasieren.	O	O	O	O	O	O	O	O	O
Das Tierschutzgesetz besagt, dass eine Tierhalterin für die Pflege des Tieres aufkommen muss. Der Tierarzt sollte die PB über ihre Pflichten dem Tier gegenüber aufklären und das Tier nur bei anschließend angeordnetem Tierhalteverbot einschläfern.	O	O	O	O	O	O	O	O	O
Die von der PB verlangte Euthanasie ist ethisch nicht vertretbar – eine kurative Behandlung ohne Bezahlung wäre ökonomisch nicht vertretbar. Der Tierarzt sollte die Euthanasie verweigern und die Familie wegschicken.	O	O	O	O	O	O	O	O	O

Das Wohl des Meerschweinchens geht vor. Der Tierarzt sollte das Tier in Obhut nehmen und behandeln, ohne die Kosten der PB anzurechnen. Anschließend soll es über einen Tierschutzverein weitervermittelt werden.	O	O	O	O	O	O	O	O	O
Es sollte möglich sein, die PB mit Argumenten zu überzeugen. Der Tierarzt sollte es mit einem Gespräch versuchen, und nur nachgeben, falls sie sich nicht überzeugen lässt.	O	O	O	O	O	O	O	O	O
Es müssen alle Bedürfnisse berücksichtigt werden. Der Tierarzt sollte deshalb der Tochter erklären, dass er ausnahmsweise das Meerschweinchen kostenlos behandeln werde, wenn die Mutter nicht bezahlen will.	O	O	O	O	O	O	O	O	O

Skalen Fall 3

Bitte denken Sie an den dritten Fall zurück:

Tierarzt Dr. Knecht-Weber wird zu einem Springpferd gerufen, das am linken Vorderbein lahmt. Nach eingehender Untersuchung ordnet er eine Behandlung und Schonung an. Der Besitzer erklärt, dass das Pferd in zwei Tagen bei einem Wettkampf

antreten soll, und fordert ihn auf, die Lahmheit mit der Gabe eines Analgetikums zu unterdrücken. Auf den Hinweis, dass eine Wettkampfbelastung unter diesen Bedingungen zu einem bleibenden Sehnenschaden führen könnte, entgegnet er, das Pferd habe eine gute Lebensversicherung.

Bitte wählen Sie die zutreffende Antwort für jeden Punkt aus:

Statement	1 (sehr schlecht	2	3	4	5	6	7	8	9 (sehr gut)
Die aufgeklärte Entscheidung liegt beim PB. Dr. Knecht- Weber kommt daher dem Wunsch des PB nach und verabreicht das Analgetikum.	O	O	O	O	O	O	O	O	O
Die Forderung des PB ist ethisch nicht vertretbar. Dr. Knecht-Weber verweigert daher ohne weitergehende Diskussion das geforderte Analgetikum.	O	O	O	O	O	O	O	O	O
Das lässt sich in einem Gespräch mit fundierten Argumenten klären. Dr. Weber- Knecht bemüht sich also, den Besitzer von der Behandlung und der Schonung des Pferdes zu überzeugen, und gibt das Analgetikum nur, wenn der PB tatsächlich nicht nachgibt.	O	O	O	O	O	O	O	O	O

Das (ökonomische und Prestige-) Interesse des PB an der Wettkampfteilnahme ist nachvollziehbar. Dr. Weber- Knecht spricht mit dem Besitzer ab, dass dies eine einmalige Ausnahme für den wichtigen Wettkampf sei und verabreicht das Analgetikum	O	O	O	O	O	O	O	O	O
Die vom PB geforderte Maßnahme verstößt gegen das Tierschutzgesetz. Dr. Knecht- Weber klärt den Besitzer über seine Pflichten gegenüber dem Tier auf und droht mit einer Anzeige beim zuständigen Veterinäramt, sollte er das Pferd beim Wettkampf starten lassen.	O	O	O	O	O	O	O	O	O

Sonstige Angaben

Wir möchten Sie abschließend um folgende Angaben bitten:

Wie Lautet Ihre Matrikelnummer?*

Welche der folgenden Lehrveranstaltungen haben Sie besucht?

Bitte wählen Sie die zutreffenden Antworten aus:

- o Vorlesung: Ethik für die Klinik
- o Vorlesung: Ethische Themen in Studium und Praxis der Tiermedizin
- o Seminar: Ethik in der Rinderpraxis
- o Seminar: Ethik in der Pferdepraxis

- Seminar: P-E-P: Kleintierpraxis
- Seminar: P-E-P: Pferdepraxis
- Seminar: P-E-P: Amtstierärzte
- Seminar: P-E-P: Schweinepraxis
- Seminar: Euthanasie in der Tiermedizin
- Seminar: Verantwortung im amtstierärztlichen Dienst
- Seminar: Tierärztliches Berufsethos: Grundlagen und Anwendungen
- Seminar: Landwirtschaftliches Praktikum auf dem LFG Ruthe
- Sonstiges:______________________________
